# Evaluation of blood culture epidemiology and efficiency in a large European teaching hospital

**DOI:** 10.1371/journal.pone.0214052

**Published:** 2019-03-21

**Authors:** R. S. Nannan Panday, S. Wang, P. M. van de Ven, T. A. M. Hekker, N. Alam, P. W. B. Nanayakkara

**Affiliations:** 1 Department of Internal Medicine, Section Acute Medicine, Amsterdam University Medical Centers, Location VU University Medical Center, Amsterdam, The Netherlands; 2 Amsterdam Cardiovascular Sciences, Amsterdam University Medical Centers, Location VU University Medical Center and Location Academic Medical Center, Amsterdam, The Netherlands; 3 Department of Epidemiology and Biostatistics, Amsterdam University Medical Centers, Location VU University Medical Center, Amsterdam, The Netherlands; 4 Department of Medical Microbiology and Infection Control, Amsterdam University Medical Centers, Location VU University Medical Center, Amsterdam, The Netherlands; University of Maryland School of Medicine, UNITED STATES

## Abstract

**Background:**

Blood cultures remain the gold standard for detecting bacteremia despite their limitations. The current practice of blood culture collection is still inefficient with low yields. Limited focus has been given to the association between timing of specimen collection at different time points during admission and their yield.

**Methods:**

We carried out a retrospective observational study by analyzing all 3,890 sets of cultures collected from the 1,962 admitted patients over the seven-month period of this study. We compared the blood culture yield between the early group (≤24 hours after admission) and the late group (> 24 hours of admission). We also investigated the effect of prehospital oral antibiotics and pre-analytical time on the first cultures in the emergency department. Epidemiology and efficiency of blood cultures were studied for each medical specialty.

**Results:**

In total, 3,349(86.1%) blood cultures were negative and 541(13.9%) were positive for one or more microorganisms. After correcting for contamination, the overall yield was 290 (7.5%). The early group (n = 1,490) yielded significantly more true-positive cultures (10.1% versus 5.8%, P<0.001) than the late group (n = 2,400). The emergency department had a significantly higher yield than general wards, 11.2% versus 5.7% (p<0.001). Prehospital oral antibiotic use and pre-analytical time did not affect the yield of first cultures at the emergency department (p = 0.735 and 0.816 respectively). The number of tests needed to obtain one true-positive culture varied between departments, ranging from 7 to 45.

**Conclusion:**

This study showed that blood cultures are inefficient in detecting bacteremia. Cultures collected during 24 hours after admission yielded more positive results than those collected later. Significant variations in blood culture epidemiology and efficiency per specialty suggest that guidelines should be reevaluated. Future studies should aim at improving blood culture yield, implementing educational programs to reduce contamination and cost-effective application of modern molecular diagnostic technologies.

## Introduction

Blood cultures (BCs) are one of the most frequently performed microbiological tests in hospitals worldwide and still remain the gold standard for detecting bacteremia [1, 2].

Currently, sepsis/septic shock and associated bloodstream infections (BSI) are among the most prevalent causes of morbidity and mortality in many European and North American countries with an estimated 157,000 deaths annually in Europe and as much as 94,000 in North America [3]. Due to its potentially life-threatening nature, physicians have a relatively low threshold to order BCs [4, 5].

Although research on the factors influencing BC yield has already led to improvements in specimen collection and reduction in contamination rates, the current practice of BC collection is still inefficient with a pathogen recovery rate of just 7% [1, 2, 5–12]. In addition, hospital protocols and guidelines advice that BCs should be collected in the event of a temperature spike in order to optimize BC yield. However, there is little evidence for this practice as previous studies have found temperature spikes to be an unreliable predictor of bacteremia [6, 13, 14].

Recently, several studies have shown that BC yield is negatively influenced by prolonged pre-analytical time [12, 15]. Pre-analytical time is defined as the time elapsed from specimen collection to incubator entry [12]. The delay in incubator insertion is mainly due to increased transport time of specimens collected in the emergency department (ED) and limited personnel and laboratory opening hours during weekends [10, 12, 15]. This can have a profound effect on BC yield and further reduces its efficiency [12, 15]. In short, the current practice of BCs is associated with reduced quality of care and increased unnecessary health care cost [5, 7, 8].

To date no studies have investigated the association between BCs collected at different time points during admission and the BC yield. If a correlation between timing of BC and BC yield exists, then this might improve the pathogen recovery rate and consequently optimize the diagnostic efficiency of BCs. Therefore, the aim of this study was to determine whether BCs collected within 24 hours after admission has higher BC yield than those collected later. In addition, we also investigated the effect of prehospital oral antibiotics and pre-analytical time on the BC yield.

## Materials and methods

### Design and setting

A retrospective observational study was conducted in the VU University Medical Center (VUmc), an academic tertiary care center in the Amsterdam metropolitan area with 733 beds. In VUmc the Medical Microbiology and Infection Prevention laboratory carries out all microbiological investigations and processes around 7,500 BCs annually. The VUmc BC protocol requires the following: (1) Strict aseptic procedures, which involves skin and BC bottle disinfection with 1% chlorhexidine for one minute before specimen collection; (2) One set of anaerobic and aerobic BC bottles are collected; (3)Blood volume is between 8–10 ml for each bottle for optimal analysis. All BCs in VUmc are processed with the BACTEC system (Becton Dickinson). This system radiometrically recognizes growth by detecting the difference in CO_2_ production (the delta) by growing bacteria over a time period [16]. It is advised that pre-analytical time is kept to a minimum in order to optimize this detection [17].

### Study population

Patients were eligible if they met the following inclusion criteria: (i) were 18 years or older; (ii) at least one BC was collected from them either at the ED or one of the general wards (all wards were included)during the seven month study period (1 September 2016 till 31 March 2017). Patients were excluded if: (i) a BC was obtained during the study period but the patient was admitted prior to the study period; (ii) a BC was obtained during the study period and the patient was discharged after the end of the study period.

### Methodology

BC data was retrieved from the electronic patient records (EPIC) [18] and central laboratory information system (GLIMS) [19]. Each set of cultures consists of an aerobic and an anaerobic bottle. The primary outcome was the yield of BCs that were obtained and sent to the laboratory within 24 hours of patient admission (early group) compared to those after 24 hours of admission (late group). Secondary outcomes were the effect of prehospital oral antibiotics as well as pre-analytical time on BC yield for ED cultures. For these values, only the first BCs collected in the ED were included. BC are collected in VUmc in all patients presenting to the ED with suspected sepsis. On the wards this is done if patients have fever (either 38.0 of 38.5 degrees Celsius). Multiple BCs per patient were included and analyzed.

Prehospital antibiotic use was assessed by attending residents in the ED via one or more of the following methods: (1) Patients were asked about antibiotic uses during anamnesis; (2) In The Netherlands the large majority of the patients are referred by the general practitioners (GP). The GPs often have an overview of patients’ current medication; (3) An overview of the medication of patients’ most recent hospital admission; (4) Pharmacies can also provide a list of current medications that patients use.

The effect of length of pre-analytical time on BC yield was assessed in three groups using dichotomous cut-off values: those in which transportation of the BC took less than 6/12/24 hours and those in which transportation took longer than 6/12/24 hours. Pre-analytical time was calculated by examining the time difference between BC collection time on EPIC and BC incubation registration time on GLIMS. Furthermore the number of BCs required to retrieve one positive culture in the different wards and the ED was also calculated and reported as number needed to draw (NND).

Each positive BC result was assessed for contamination (false-positive) according to established criteria [9]. Thereafter, all false-positive cultures were coded as negative cultures. A list of microorganisms that are associated with false-positive results were derived from a review on this subject [9], the following microorganisms were considered as contaminants: Micrococcus species, Bacillus species other than B. anthracis, Coagulase-negative staphylococci (CoNS), Corynebacterium species, Propionibacterium acnes. An overview of these species can be found in S1 Appendix. In accordance with the VUmc medical research regulations, this study was approved by the Medical Ethical Committee of the VU University Medical Center (METC VUmc). The METC VUmc waived the requirement for informed consent.

### Statistical analysis

Descriptive statistics were used to describe patient characteristics, presented as frequency (proportion), mean ± SD or as median [IQR]. Difference in BC yield (proportion positive and proportion true-positive) between the early group and the late group, was compared using generalized estimating equation (GEE) analysis with an exchangeable correlation structure to account for within-patient dependence of repeated BCs. To correct for possible confounding variables we adjusted the GEE models by including main effects for the candidate confounders to the linear predictor. Chi-squared test was used for dichotomous outcomes that were only obtained once for each patient.

With the available sample size of 1962 patients, the minimum difference detected would be 3.6% with a power of 80% in true-positive cultures between the early group and the late group, assuming two-sided testing at an overall 5% significance level.

A p<0.05 was considered as being statistically significant. All analyses were performed in IBM SPSS Statistics 22.0 (Chicago, USA).

## Results

### Patient characteristics

**Fig 1:**
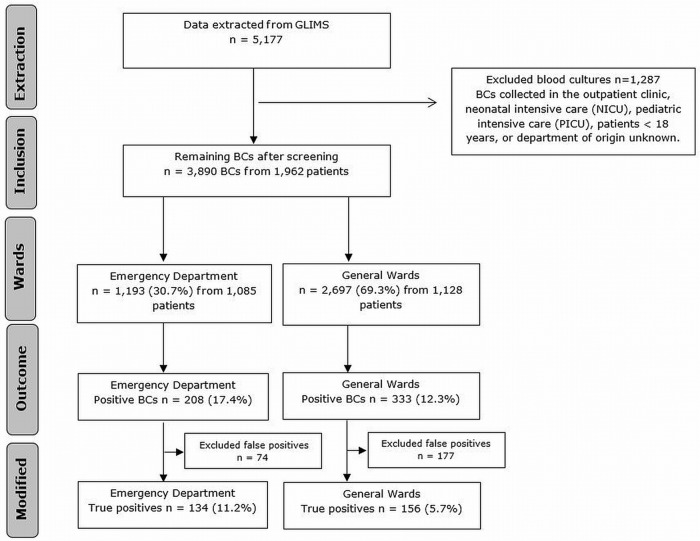
Flowchart of blood culture yield. BC = blood culture.

A total of 5,177 BCs were assessed for eligibility during the study period. Fig 1 illustrates the selection of BCs included in this study and BC yield. In total, 3,890 BCs collected from 1,962 patients fulfilled all the required criteria. Of those patients the majority was male (57%) and median age was 65 years (IQR: 52–75). The median number of BCs collected per patient per admission was 1.0 (IQR 1.0–2.0). The median time until the collection of the first BC was 19.7 hours (IQR 13.6–45.7).

### Blood culture results

Of the 3,890 BCs, 3,349 (86.1%) were negative and 541 (13.9%) were positive for one or more microorganisms. Of the 541 positive BCs, there were 251 (46.4%) contaminations, meaning that 290 blood cultures were true-positive (7.5% of all blood cultures). Thus, the overall contamination rate in this study is 6.4%. Table 1 compares the BC yield in the early and the late group. 1,490 BCs (38.3%) were collected in the early group (male 57.9%, female 42.1%), while 2,400 (61.7%) were collected in the late group (male 56.5%, female 43.5%). The mean age of the patient in the early group and the late group was similar (62.0 ± 17.3 years versus 62.1 ± 18.2 years). BCs collected at an early moment were more often true-positive (p<0.001) (10.1% versus 5.8%), even after correcting for age and gender. In the early group, 1193 BCs were collected in the ED and 297 BCs in the general wards. In the late group, 120 BCs were collected in the ED and 2280 BCs in the general wards. As the vast majority of BCs in the early group were collected in the ED, we adjusted for ED in our analysis. After adjusting for department of collection (ED compared to general wards), no difference was found between the early and late group in true-positive yield.

**Table 1:** Combined emergency department and general wards blood culture yield: ≤ 24 hours (early group) versus > 24 hours (late group).

Combined emergency department & general wards	BC n(%)	True positive n(%)	NND	OR (95% CI)	P-value
≤ 24 hours	1,490 (38.3%)	151 (10.1%)	10	2.09 (1.54–2.83)	*<0*.*001*
> 24 hours	2,400 (61.7%)	139 (5.8%)	18		

BC = blood culture. NND = number needed to draw. OR = odds ratio. CI = Confidence interval

The NND in the early group was 10 and that of the late group 18. A comparison between the ED and general wards showed that BCs collected in the ED had a significantly higher BC true-positive yield, even after adjustment for age and gender (11.2% and 5.8%, p<0.001).

### Effect of prehospital antibiotics on blood culture yield

In 1,085 ED patients the effect of prehospital oral antibiotics on the first BC outcome was assessed. Table 2 shows the number of patients using prehospital oral antibiotics and BC yields per group. 208 patients (19.1%) used prehospital oral antibiotics at the time of admission, 772 (71.2%) did not and of the remaining 9.7% this information was missing. Those who had used prehospital oral antibiotics demonstrated in 10.1% of the cases a true-positive BC while those who did not use prehospital oral antibiotics had a true-positive BC in 11.0% of the cases (p = 0.735).

**Table 2:** The effect of prehospital oral antibiotic use on blood culture yield in the emergency department.

Antibiotics prior to admission	Patients n(%)	True positive BC n(%)	P-value
Yes	208 (19.1%)	21 (10.1%)	**0.735**
No	772 (71.2%)	85 (11.0%)	
Unknown	105 (9.7%)	12 (11.4%)	
Total	1,085		

BC = blood culture

### Effect of pre-analytical time on blood culture yield

In 1,085 ED patients the effect of pre-analytical time on BC yield was analyzed. The median pre-analytical time was 15 hours (IQR: 9–19). No significant difference was found using three predefined cut-off points (≤ 6 hours versus > 6 hours, ≤ 12 hours versus > 12 hours, ≤ 24 hours versus > 24 hours), with p-values of 0.816, 0.474, and 0.676 respectively (Table 3).

**Table 3:** The Impact of Pre-analytical Time on blood culture yield.

Pre-analytical time	Blood cultures n(%)	True positive n(%)	P-value
≤ 6 hours	212 (19.5%)	22 (10.4%)	**0.816**
> 6 hours	873 (80.5%)	96 (11.0%)	
≤ 12 hours	432 (39.8%)	51 (11.8%)	**0.474**
> 12 hours	653 (60.2%)	67 (10.3%)	
≤ 24 hours	996 (91.8%)	107 (10.7%)	**0.676**
> 24 hours	89 (8.2%)	11 (12.4%)	

### BC epidemiology and efficiency per department

The ED, hematology, intensive care unit (ICU) and acute medical unit (AMU) were the departments that most frequently ordered BCs, representing 30.7%, 13.9%, 12.2% and 7.3% respectively. Analysis of the number of BCs that was required to find one true-positive culture (NND) differed greatly per department. Table 4 illustrates the epidemiology of BCs by department. The ED and the short stay unit (SSU) were the most efficient at this process, with a NND of 9 and 7, respectively, while the neurosurgical department, AMU and obstetrics department performed the poorest in this respect, with NNDs of 45, 29 and 28, respectively.

**Table 4:** Number of blood cultures, BC yield, contamination rate, and NND per department.

Department	BC n (%)	Positive n (%)	True Positive n (%)	False Positive n (%)	NND
ED^a^	1,193 (30.7%)	208 (17.4%)	136 (11.4%)	72 (6.0%)	9
Hematology	540 (13.9%)	70 (13.0%)	32 (5.9%)	38 (7.1%)	17
ICU^b^	476 (12.2%)	77 (16.2%)	19 (4.0%)	58 (12.2%)	26
AMU^c^	283 (7.3%)	21 (7.4%)	10 (3.5%)	11 (3.9%)	29
Surgery	190 (4.9%)	19 (10.0%)	11 (5.8%)	8 (4.2%)	18
Oncology	160 (4.1%)	16 (10.0%)	9 (5.6%)	7 (4.4%)	18
CCU^d^	156 (4.0%)	26 (16.7%)	17 (10.9%)	9 (5.8%)	10
ANG/URO/NEPH^e^	155 (4.0%)	21 (13.5%)	12 (7.7%)	9 (5.8%)	13
Internal Medicine	136 (3.5%)	13 (9.6%)	7 (5.1%)	6 (4.5%)	20
Cardiology	119 (3.1%)	16 (13.4%)	12 (10.1%)	4 (3.3%)	10
Neurology	117 (3.0%)	11 (9.4%)	6 (5.1%)	5 (4.3%)	20
MCU^f^	110 (2.8%)	22 (20.0%)	6 (5.5%)	16 (14.5%)	19
Pulmonology	80 (2.1%)	6 (7.5%)	3 (3.8%)	3 (3.7%)	27
Neurosurgery	45 (1.2%)	3 (6.7%)	1 (2.2%)	2 (4.5%)	45
ENT^g^	44 (1.1%)	5 (11.4%)	5 (11.4%)	0 (0.0%)	9
Gynecology	37 (0.9%)	1 (2.7%)	0 (0.0%)	1 (2.7%)	-
Obstetrcs	28 (0.7%)	1 (3.6%)	1 (3.6%)	0 (0.0%)	28
SSU^h^	21 (0.5%)	5 (23.8%)	3 (14.3%)	2 (9.5%)	7

BC = blood culture. NND = number needed to draw.

^a^Emergency Department

^**b**^Intensive Care Unit

^**c**^Acute Medical Unit

^**d**^Coronary Care Unit

^**e**^Angiology/Urology/Nephrology

^**f**^Medium Care Unit

^**g**^Ear/Nose/Throat

^**h**^Short Stay Unit.

## Discussion

This study has shown major differences in BC outcomes in the ED and general wards in relation to the timing of BCs after admission Specimens collected and incubated within the first 24 hours of admission had a significantly higher BC yield compared to those that were obtained after the first 24 hours of admission. This effect may have been caused by the large number of true-positive BCs in the ED. To the best of our knowledge, this is the first study to evaluate the relationship between timing of BCs during admission and BC yield.

BC guidelines recommend BCs to be collected before administering antibiotics. In our study, prehospital oral antibiotic use had no significant effect on the BC yield of the first cultures in the ED. This may partially due to strict regulations of antibiotics and well established antibiotic stewardship in The Netherlands [20]. Most patients who were on antibiotics at the time of ED arrival used narrow spectrum antibiotics (e.g. feneticillin, flucloxacillin and nitrofurantoin) obtained from their general practitioners [20]. It is impossible to retrieve the exact time of BC collection on the wards due to the discrepancy between BC order time and BC collection time. When the order for the BC collection on the ward is placed in the electronic patient file, only the BC order time is recorded, but the time of BC collection can be hours later and the time of BC collection itself is not recorded. Therefore we were unable to determine the effect of inpatient antibiotic administration on the outcome of BCs drawn in the wards. It might be possible that the inpatient antibiotic administration led to the lower yield in wards and after 24 hours. This should be investigated in future studies.

International guidelines have not reached consensus on the time frame for BC transportation [21–23]: current recommendations vary from within two to four hours after collection [21–23]. Venturelli and colleagues (2017) investigated the effect of pre-analytical time on BC yield in the University Hospital of Modena in Italy by analyzing 50,955 BCs collected from 7,035 patients with sepsis. This study concluded that longer pre-analytical time (≤ 2 hours versus > 2 hours) is associated with a decreased pathogen recovery rate [12]. In contrast, our study showed that pre-analytical time for the three predefined cut-off values had no influence on BC yield. Our study populations, however, were significantly different. Venturelli et al. analyzed cultures collected from septic patients, whereas we studied all first BCs collected in the ED for pre-analytical time. Moreover, contrary to the Italian study where the laboratory in the university hospital is closed on Sundays and holidays, the microbiology laboratory in VUmc also operates on weekends and holidays. This drastically reduces the extreme values of pre-analytical time which can result from closed facilities over the weekend.

The overall result of the BCs in this study is in line with previous research where the majority of the cultures were negative [1, 2, 5–12]. Previous studies have developed algorithms consisting of vital parameters and lab values to increase BC yield [24, 25], however these algorithms differ per focus of infection and might be time consuming due to their complex nature.

In our study, the overall contamination rate was 6.5%, which is higher than the desired contamination rate of 2–3%. Earlier studies have demonstrated an association between high contamination rate and university teaching hospitals [7, 26]. Despite the strict aseptic procedures in VUmc, contamination rate is comparable to that of other similar sized European hospitals, where rates could be as high as 7.4% [26]. Some improvements could be achieved by implementing an educational program for nurses or by deploying a specialized phlebotomy team [7, 27, 28]. These educational programs should also include instructions regarding the amount of blood volume that should be collected, as underfilling of these bottles reduces sensitivity and increases contamination rate [29]. However, long term effect of these measures remains unclear.

This study has several strengths. As far as we know, this is the first study to evaluate the epidemiology and clinical significance of BCs in the general hospital population. A previous study conducted by Morton et al. [15] researched the relationship between BC yield and focus of the infection, however this study was only conducted in critical care unit patients.

Our study analyzed BC epidemiology and significance per department by analyzing various parameters including BC yield, contamination rate, and NND in the general hospital population. This provides a novel insight into the diagnostic value and clinical relevance of BCs for each medical specialty. The dramatic differences in NND suggest that BCs are efficient and valuable in some departments while remarkably inefficient in others. To the best of our knowledge, this is the first attempt to evaluate the efficiency of BCs using NND. In some departments, NNDs were as high as 45. This raises a question on the diagnostic value of BCs for certain medical specialties, as they may benefit from exploring alternative and potentially more sensitive molecular diagnostic tests such as polymerase chain reaction (PCR) and mass spectrometry for the detection of bacteremia/fungemia. [30, 31]. Previous studies have already shown promising results from these techniques regarding sensitivity and rapid identification of organisms with a low contamination rate. As the technology advances, these techniques will increasingly become more cost-effective when compared to conventional BC and therefore implementation should be considered [32, 33].

The long study period of seven months enabled us to include a large number of patients of all medical specialties with a wide-range of clinical conditions. We included patients at the ED and general wards who had at least one BCs taken during their admissions. We evaluated the effect of pre-analytical time on the BC yield by analyzing the first cultures collected during their ED stay. The specimen collection time on the electronic medical record is only trustworthy for ED admissions as blood is collected immediately after an order was given.

Despite these strengths, there also some limitations. Firstly, this study was a retrospective analysis of electronically stored data and therefore it was not possible to evaluate the correlation between BC outcome and clinical status of the patient. To minimize such effect, we included all BCs during the study period except those from pediatric patients due to ethical considerations. Secondly, this was a single center study representing a relatively large university teaching hospital. Thirdly, a few microorganisms that were considered to be contaminations in this study, including *Staphylococcus epidermidis* could potentially be pathogenic in patients with prosthetics or other artificial materials. We do not know how many of these patients had central venous catheters in place (PICC, Port, etc) and where the BC was obtained, this may have also affected the yield and interpretation. However, due to limited research on the complex interaction between coagulase-negative staphylococci and prosthetic materials, it remains unknown what the percentage is of these microorganisms that can lead to BSI in this subgroup of patients [34].

## Conclusion

Blood cultures collected and incubated within 24 hours of admission had a higher yield compared to those after 24 hours of admission. Although this effect might be due to the high yield in the ED within the first 24 hours of admission, we advise physicians to be critical when ordering BCs, especially after 24 hours of admission. Pre-analytical time and prehospital oral antibiotic use had no effect on BC yield. This study shows that blood cultures are inefficient in detecting bacteremia. The significant variations in epidemiology and efficiency per specialty suggest that BC protocols should be reevaluated and adjusted per department. Future studies should aim at improving BC yield, implementation of educational programs on BC collection and the application of modern molecular diagnostic techniques as they increasingly become more cost-effective.

## Supporting information

S1 Appendix List of positive culture classifications: True positive and contaminant microorganisms. *Possible true infection in patients with prosthetic devices and central venous catheters.
(DOCX)
