# Supplementary material for: Evaluation of blood culture epidemiology and efficiency in a large European teaching hospital
Source: PLoS One. 2019 Mar 21;14(3):e0214052. doi: 10.1371/journal.pone.0214052 (PMC6428292; doi:10.1371/journal.pone.0214052)
Supplement: S1 Appendix — *Possible true infection in patients with prosthetic devices and central venous catheters. (DOCX) [file pone.0214052.s001.docx]

| **True positives** | **Contaminants** |
| --- | --- |
| Staphylococcus aureus | Micrococcus species |
| Streptococcus pneumoniae | Bacillus species other than B. anthracis |
| Escherichia coli & other Enterobacteriaceae | Coagulase-negative staphylococci (CoNS)* |
| Enterococci | Corynebacterium species* |
| Viridans group streptococci | Propionibacterium acnes* |
| Clostridium perfringens |  |
| Pseudomonas aeruginosa |  |
| Streptococcus pyogenes |  |
| Streptococcus agalactiae |  |
| Listeria monocytogenes |  |
| Neisseria meningitidis |  |
| Neisseria gonorrhoeae |  |
| Haemophilus influenzae |  |
| Bacteroides fragilis |  |
| Candida species |  |
| Cryptococcus neoformans |  |

*Possible true infection in patients with prosthetic devices and central venous catheters
